# Evaluation of an ethidium monoazide–enhanced 16S rDNA real-time polymerase chain reaction assay for bacterial screening of platelet concentrates and comparison with automated culture

**DOI:** 10.1111/trf.12256

**Published:** 2013-05-23

**Authors:** Jeremy A Garson, Poorvi Patel, Carl McDonald, Joanne Ball, Gillian Rosenberg, Kate I Tettmar, Susan R Brailsford, Tyrone Pitt, Richard S Tedder

**Affiliations:** National Transfusion Microbiology Laboratories, NHSBT/HPA Epidemiology Unit, NHS Blood and TransplantColindale, London; Research Department of Infection, Division of Infection and Immunity, University College LondonLondon; Blood Borne Virus Unit, Viral Reference Department, Centre for Infections, Health Protection AgencyColindale, London, UK

## Abstract

**BACKGROUND:**

Culture-based systems are currently the preferred means for bacterial screening of platelet (PLT) concentrates. Alternative bacterial detection techniques based on nucleic acid amplification have also been developed but these have yet to be fully evaluated. In this study we evaluate a novel 16S rDNA polymerase chain reaction (PCR) assay and compare its performance with automated culture.

**STUDY DESIGN AND METHODS:**

A total of 2050 time-expired, 176 fresh, and 400 initial-reactive PLT packs were tested by real-time PCR using broadly reactive 16S primers and a “universal” probe (TaqMan, Invitrogen). PLTs were also tested using a microbial detection system (BacT/ALERT, bioMérieux) under aerobic and anaerobic conditions.

**RESULTS:**

Seven of 2050 (0.34%) time-expired PLTs were found repeat reactive by PCR on the initial nucleic acid extract but none of these was confirmed positive on testing frozen second aliquots. BacT/ALERT testing also failed to confirm any time-expired PLTs positive on repeat testing, although 0.24% were reactive on the first test. Three of the 400 “initial-reactive” PLT packs were found by both PCR and BacT/ALERT to be contaminated (*Escherichia coli*, *Listeria monocytogenes*, and *Streptococcus vestibularis* identified) and 14 additional packs were confirmed positive by BacT/ALERT only. In 13 of these cases the contaminating organisms were identified as anaerobic skin or oral commensals and the remaining pack was contaminated with *Streptococcus pneumoniae*.

**CONCLUSION:**

These results demonstrate that the 16S PCR assay is less sensitive than BacT/ALERT and inappropriate for early testing of concentrates. However, rapid PCR assays such as this may be suitable for a strategy of late or prerelease testing.

Transfusion-transmitted bacterial infection remains an unresolved problem associated with significant morbidity and mortality.^1^ Bacterial contamination of platelets (PLTs) is responsible for most cases of blood component associated sepsis and globally the reported prevalence of such contamination ranges from 0.03% to 0.7%.^2^,^3^ PLTs pose the greatest risk because, to preserve their function, they are stored at 22 to 24°C with constant agitation. These storage conditions provide for the majority of contaminating bacteria an excellent growth environment, so that small numbers of organisms may rapidly increase to levels likely to cause sepsis.^4^

In an effort to address this problem many countries have introduced bacterial screening of PLT concentrates by culture-based methods. Automated culture systems such as the BacT/ALERT 3D microbial detection system (bioMérieux UK Ltd, Basingstoke, Hampshire, UK) are widely used for this purpose.^5^ However, the use of culture systems may result in the transfusion of a PLT component before the determination of a positive result.^6^ This is particularly likely with slow-growing organisms and very low initial bacterial loads.^7^,^8^ Despite the use of such culture methods, and the introduction of improved skin decontamination techniques and diversion pouches,^6^ the residual risk of PLT transfusion-transmitted sepsis is estimated to be in the region of 1 in 45,000 to 90,000.^7^,^9^

Recognition of the limitations of slow, culture-based screening methods has encouraged the development of more rapid detection technologies including immunologic assays (PGD, Pan Genera Detection system, Verax Biomedical, Inc., Marlborough, MA), flow cytometry (BactiFlow, AES Chemunex GmbH, Bruchsal, Germany), and polymerase chain reaction (PCR) amplification of bacterial nucleic acid.^10^ The strengths and weaknesses of these alternative technologies have been reviewed by others^10^–^12^ and key performance characteristics are summarized in Table 1. Although PCR-based methods may be more technically demanding than alternative rapid detection techniques they potentially offer assays with greater sensitivity.

**Table 1:** Key features of assays available for detecting bacterial contamination of PLT concentrates (data derived from publications^10^–^13^)

Assay type	Sensitivity CFUs/mL*	Assay time	Sample volume (mL)	Technical complexity†	Early testing strategy‡	Late testing strategy§
BacT/ALERT culture system	1-10	1-7 days	8-16	Medium	Yes	No
PGD (Verax) immunoassay	10^4^-10^6^	1.5 hr	0.5	Low	No	Yes
BactiFlow flow cytometry	>150	1 hr	1	Medium	No	Yes
PCR nucleic acid amplification	10-30	3-4 hr	0.2-2	High	?	Yes

* Approximate ranges; sensitivity varies according to bacterial species, assay protocol, and so forth.

† Technical complexity is associated with requirement for highly skilled laboratory personnel.

‡ Potential suitability of assay for early testing after preincubation period and release on a negative-to-date basis.

§ Potential suitability of assay for late testing strategy (e.g., prerelease testing).

The most widely used target for PCR assays is the highly conserved 16S ribosomal RNA gene (16S rDNA) which is present as multiple copies in all bacterial genomes.^14^,^15^ Theoretically, PCR-based assays should be capable of detecting single molecules of 16S rDNA and therefore achieve extremely high sensitivity but this potential has not generally been realized. The main reason for the disappointing performance of most 16S PCR assays is the ubiquitous presence of low-level contaminating bacterial DNA in laboratory consumables and PCR reagents, particularly in *Taq* polymerases.^16^

Numerous approaches to PCR reagent decontamination have been described^17^–^21^ but most have proven ineffective or unreliable in subsequent studies.^22^,^23^ We recently compared the effectiveness of several different reagent decontamination techniques and found that all but one of them failed to achieve the level of decontamination required. The single successful method employed ethidium monoazide (EMA) treatment of the PCR master mix followed by photoactivation, which totally eliminated bacterial DNA contamination without compromising assay sensitivity. The optimized assay was shown to be capable of detecting a wide range of organisms known to be associated with contamination of PLT concentrates, at levels down to approximately 1 colony-forming unit (CFU)/mL.^24^ In this study, we evaluate the performance of this EMA-enhanced real-time 16S rDNA PCR for screening of PLT concentrates in parallel with BacT/ALERT automated culture. In February 2011, National Health Service Blood and Transplant (NHSBT) implemented the screen testing of all PLT components using the BacT/ALERT system.^25^

## MATERIALS AND METHODS

### NHSBT bacterial screening procedure

PLT components were sampled between 36 and 48 hours from collection. Aerobic and anaerobic culture was performed with 8 mL inoculated per culture bottle. Bottles were incubated at 36°C for the remainder of the 7-day shelf life of the component. All initial-reactive bottles and components were sent to NHSBT National Bacteriology Laboratory for confirmatory and reference work.

### Time-expired PLTs

Leukoreduced PLTs were prepared according to standard NHSBT procedures. Of the 2050 time-expired PLTs tested 745 were pooled and buffy coat derived and 1305 were apheresis components. Pooled PLTs were prepared by pooling the buffy coats from four whole blood donations and resuspending in approximately 250 mL of male donor plasma. CPD anticoagulant was used and PLT concentrates were stored at 22°C with agitation. For apheresis PLTs the anticoagulant used was ACD. Each unit contained more than 2.4 × 10^11^ PLTs and fewer than 5 × 10^6^ white blood cells. PLT concentrates were defined as “time-expired” at 7 days postdonation (note that before the introduction by NHSBT of BacT/ALERT screening the shelf-life for PLTs was 5 days). The 2050 time-expired PLT concentrates were tested by EMA-enhanced 16S rDNA real-time PCR assay and in parallel by the BacT/ALERT automated culture system. All time-expired PLTs were collected from blood donors in England and processed and manufactured at NHSBT sites in Bristol, Sheffield, Manchester, Brentwood, and London (Colindale) during the period July 2010 to October 2011.

### Fresh PLTs

In addition to the 2050 time-expired PLTs, 176 “fresh” PLTs (i.e., prepared and tested on the day of donation) were also tested by EMA-enhanced 16S rDNA real-time PCR assay. All fresh PLTs were donated in England and processed by NHSBT at the Colindale site between March and April 2012.

### Initial-reactive PLTs

The term “initial reactive” is used in this study to refer to PLTs that were flagged as reactive by BacT/ALERT on initial testing at NHSBT laboratories in Bristol, Sheffield, Manchester, Newcastle, and Colindale during the period November 2011 to February 2012. Four-hundred initial-reactive PLT packs were transferred at 4°C to the NHSBT National Bacteriology Laboratory at Colindale for confirmatory and reference work, which included retesting by BacT/ALERT automated culture and for analysis by EMA-enhanced 16S rDNA real-time PCR.

### Automated nucleic acid extraction

Nucleic acid was extracted from PLT concentrates using an automated high-throughput platform (MagNA Pure 96 system with MagNA Pure 96 DNA and Viral NA small volume kit reagents, Roche Diagnostics Ltd, Burgess Hill, West Sussex, UK) previously shown to be capable of producing uncontaminated extracts.^24^ Manipulations were performed in a Class II biological safety cabinet and sterile disposable plasticware (aerosol-resistant, DNA-free pipette tips, Rainin BioClean GP-10F and GP-20F, Anachem Ltd, Bedfordshire, UK) was employed throughout. Two milliliters of PLT concentrate was centrifuged at 10,000 × *g* for 5 minutes and the pellet was resuspended in 200 μL of supernatant. The resuspended pellet was then frozen at −20°C and rethawed at room temperature before nucleic acid extraction on the MagNA Pure 96 platform as per manufacturer's instructions. The elution volume was 50 μL. A second 2-mL aliquot of each PLT concentrate was also centrifuged as described above and the resuspended pellet stored frozen at −20°C to be used to confirm any positive result arising from PCR testing of the first aliquot.

### EMA-enhanced 16S rDNA real-time PCR assay

Universal primers and TaqMan probe were used to amplify and detect a highly conserved 160-bp region of the bacterial 16S rDNA gene in a 25-μL multiplex PCR also containing internal control human mitochondrial DNA (mtDNA) primers and probe. Full sequence details of the primers (16S-F, 16S-R, Propioni-F, Bacteroides-R, mtDNA-F, mtDNA-R) and probes (16S-Probe, mtDNA-Probe) have been published previously.^24^ All oligonucleotides were synthesized by Applied Biosystems (Warrington, UK). PCR master mix contained 1× PCR buffer (Qiagen, Crawley, West Sussex, UK; HotStarTaq buffer), 3.0 mmol/L MgCl_2_, 0.2 mmol/L each dNTP (Invitrogen, Life Technologies Ltd, Paisley, UK), 400 nmol/L each 16S-F and 16S-R, 200 nmol/L 16S-Probe, 300 nmol/L Propioni-F, 200 nmol/L Bacteroides-R, 20 nmol/L each mtDNA-F and mtDNA-R, 100 nmol/L mtDNA-Probe, 0.625 U HotStarTaq polymerase (Qiagen) per 20 μL, and 1.2 μmol/L EMA (Biotium, Inc., Hayward, CA). All manipulations involving EMA, before and including the photoactivation step, were performed in a dark room under red safelight illumination as described previously.^24^ The PCR master mix was placed on ice in a closed polypropylene microtube (Sarstedt Ltd, Leicester, UK; catalogue no. 72.692.005) and exposed to a 500-W halogen light source (bulb type R7s, Powerlight Ltd, Leeds, UK) for 3 minutes at a distance of 20 cm. After photoactivation, the EMA-treated PCR master mix was aliquoted into the wells (20 μL per well) of a 96-well optical plate (Applied Biosystems, catalogue no. 4306737) and 5 μL of extracted DNA template was added to each well. Thermal cycling was performed on a real-time PCR system (ABI 7500, Applied Biosystems) using the following thermal profile: 15 minutes at 95°C to activate the HotStarTaq, followed by 40 cycles of 94°C for 30 seconds, and 60°C for 35 seconds. Three negative controls with EMA-treated PCR master mix and no added template were included in each run. Unsheared *Escherichia coli* DNA (Sigma-Aldrich Ltd, Poole, Dorset, UK; catalogue no. D4889) diluted in 0.1% Triton X-100 was included as a positive control in each run at dilutions corresponding to approximately 5000, 500, 50, and five genome copies per PCR procedure.

### BacT/ALERT automated culture and bacterial identification

The BacT/ALERT continuous monitoring system, based on the detection of CO_2_ produced by bacteria, was used according to manufacturer's instructions. Bottles were inoculated with 8-mL aliquots of PLT concentrates and incubated at 36°C in both aerobic and anaerobic conditions until the end of the PLT shelf life, that is, 7 days postdonation. Identification of positive BacT/ALERT cultures was performed using conventional bacteriologic techniques.

## RESULTS

### Sensitivity of PCR assay and threshold setting for PLT screening

**Figure 1:**
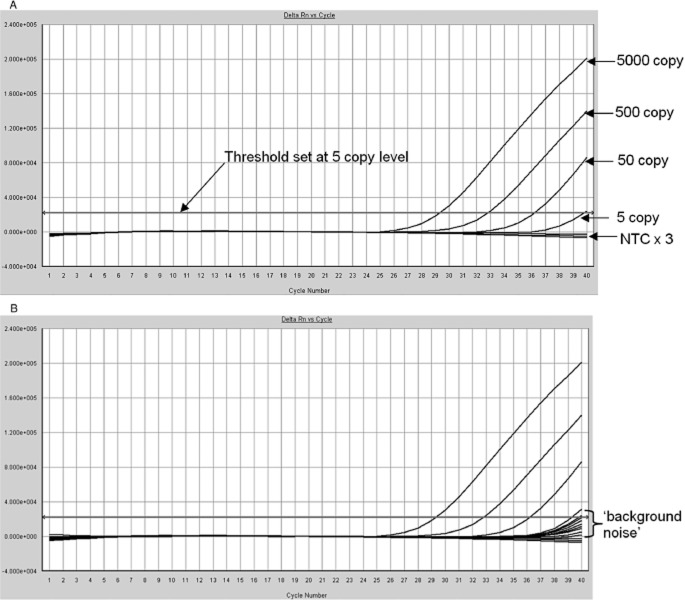
(A) Typical dilution series of 5000, 500, 50, and five *E. coli* genome copies per PCR procedure and three NTCs. Threshold in this case was set at the subsequently abandoned five-copy level. The horizontal axis indicates cycle number and the vertical axis indicates fluorescence (Delta Rn). (B) The same experimental run as illustrated in A but with curves from eight PLT concentrates added. Note typical spreading fan of late amplification curves, one of which crosses the five-copy threshold. The horizontal axis indicates cycle number and the vertical axis indicates fluorescence (Delta Rn).

The sensitivity of the EMA-enhanced 16S rDNA real-time PCR was confirmed in each assay run with a 10-fold dilution series of *E. coli* DNA representing 5000, 500, 50, and five genome copies per reaction. Typical results are illustrated in Fig. 1A, which shows that five genome copies are readily detected and clearly distinguishable from the three no-template controls (NTCs). For initial experiments the threshold was set at a level that was intersected by the five-genome-copy control at 40 cycles. However, it soon became apparent that this five-genome-copy threshold level was too low for screening PLTs because a significant proportion of them contained bacterial DNA at or around this level, as illustrated in Fig. 1B. The spreading fan of late amplification curves referred to as “background noise” in Fig. 1B was seen in all runs with time-expired PLTs (2050 samples tested in 23 separate PCR procedures) and the same phenomenon was observed on testing the 176 fresh PLT packs. Approximately 7% of all PLT packs tested contained bacterial DNA at a concentration that crossed the five-genome-copy threshold level, typically between Cycle 38 and Cycle 40 (equivalent to between 20 and five genome copies per reaction). In contrast, amplification signals were not seen with NTCs or with extraction buffer–negative controls extracted in parallel through the MagNA Pure 96 system.

In view of these findings the threshold for screening PLTs was raised to a level slightly higher than that of the “background noise.” The threshold was therefore set in each PLT screening run so that it was intersected by the 50-genome-copy control at Cycle 39 (equivalent to a detection limit of 25 genome copies per reaction in a 40-cycle PCR procedure). This modified threshold level was used for the analysis of all the PCR data presented in this study.

### Comparison of PCR and BacT/ALERT results on time-expired PLT concentrates

All time-expired PLTs were tested by EMA-enhanced 16S rDNA real-time PCR and in parallel by the BacT/ALERT automated culture system. On first testing by PCR, 23 (1.12%) of the 2050 time-expired PLT concentrates generated a positive 16S signal, that is, above the modified threshold level, with threshold cycle (Ct) values between Cycle 38 and Cycle 40. Only seven (0.34%) of these were found to be positive on repeat PCR testing of the first nucleic acid extract, also with Ct values between 38 and 40. However, none of the seven was subsequently confirmed positive by reextraction and PCR testing of frozen stored second aliquots. BacT/ALERT testing of the 2050 time-expired PLT concentrates also failed to find any which were confirmed positive on repeat testing although 0.24% were reported reactive on the first test.

### Comparison of PCR and BacT/ALERT results on initial-reactive PLT concentrates

**Figure 2:**
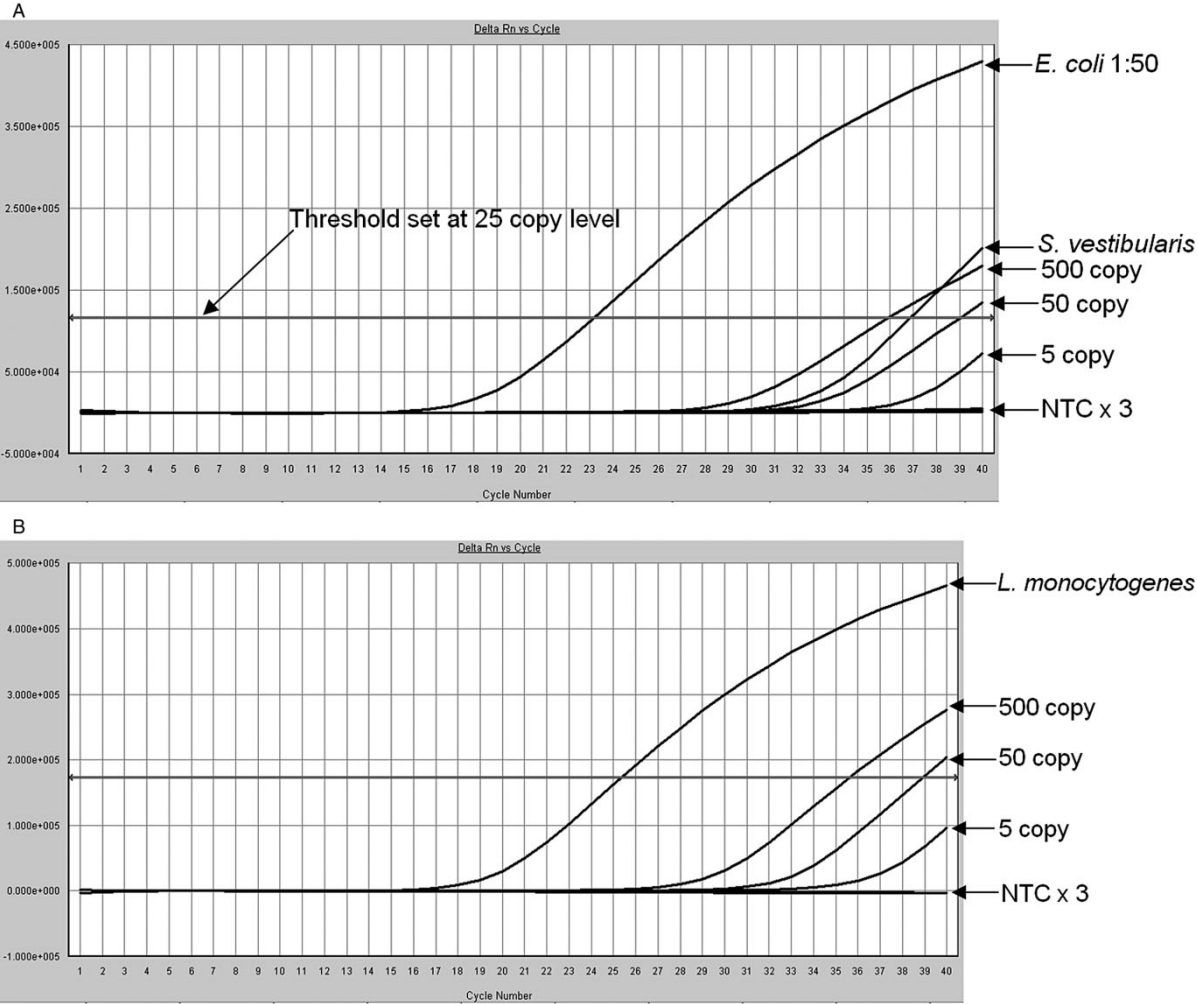
(A) Amplification curves from two of the three contaminated initial-reactive PLT concentrates (retesting of the first PCR extracts). Samples from the *E. coli*-contaminated pack at 1:50 dilution and from the *S. vestibularis*-contaminated pack are shown. The 500-, 50-, and five-copy standards are also illustrated together with three NTCs. The horizontal axis indicates cycle number and the vertical axis indicates fluorescence (Delta Rn). (B) The *L. monocytogenes*-contaminated PLT concentrate exhibiting a Ct of approximately 25 cycles on testing of the second nucleic acid extract. The horizontal axis indicates cycle number and the vertical axis indicates fluorescence (Delta Rn). Note that the threshold is set at the 25-copy level.

Four-hundred initial-reactive PLT packs were transferred from regional testing centers to Colindale for testing by both methods. Twelve of the 400 initial-reactive PLTs gave a positive 16S signal on first testing by PCR and three of the 12 were also positive on repeat testing of the first nucleic acid extract. The Ct values of the remaining nine samples that were not confirmed positive on repeat testing of the first extract were within the range 38 to 40 cycles on initial PCR testing. The three repeat-reactive samples were confirmed 16S PCR positive after reextraction of the frozen stored second aliquots (Fig. 2). The same three initial-reactive PLT concentrates were also confirmed positive by BacT/ALERT on testing and retesting, the contaminating bacteria being identified as *E. coli*, *Streptococcus vestibularis*, and *Listeria monocytogenes* (Table 2).

**Table 2:** Details of bacterially contaminated PLT packs detected by 16S rDNA PCR and/or by BacT/ALERT testing

Number of packs*	Organism isolated	BacT/ALERT result (incubation time†)	16S rDNA PCR result (Ct)
1	*E. coli*	Positive (3.6 hr)	Positive (23 cycles‡)
1	*L. monocytogenes*	Positive (7.3 hr)	Positive (25 cycles)
1	*S. vestibularis*	Positive (10 hr)	Positive (37 cycles)
6	*P. acnes*	Positive (74-132 hr)	Negative
3	*S. saccharolyticus*	Positive (54-65 hr)	Negative
1	*S. salivarius*	Positive (14.3 hr)	Negative
1	*S. oralis*	Positive (13.5 hr)	Negative
2	Coagulase-negative *Staphylococcus*	Positive (18-25 hr)	Negative
1	*S. pneumoniae*	Positive (14.2 hr)	Negative

* Note that none of the PLT packs listed in this table were actually transfused.

† Incubation time = time until positive reaction signaled.

‡ Ct of 23 cycles obtained with *E. coli*-contaminated sample diluted 1:50.

An additional 14 initial-reactive PLT concentrates were confirmed positive by BacT/ALERT testing only (Table 2). In 13 of these cases the contaminating organisms were identified as anaerobic skin or oral commensals including *Propionibacterium acnes* (n = 6) and *Streptococcus oralis*. In the remaining case the contaminating organism, detected after 14.2-hour BacT/ALERT incubation, was identified as *Streptococcus pneumoniae*. The failure of the 16S PCR to detect this organism was found not to be due to sequence mismatch because the cultured isolate was readily detected in subsequent experiments (data not shown). Furthermore, the failure of the 16S PCR to detect *P. acnes, Staphylococcus saccharolyticus, Streptococcus salivarius, S. oralis*, and coagulase-negative *Staphylococcus* in these PLT packs was not due to sequence mismatch since BLAST analysis revealed perfect alignment of the oligonucleotide primers and probes with all these organisms. Note that all 17 repeat-positive BacT/ALERT results were confirmed by culturing the same organism from the original PLT pack and the initial-reactive BacT/ALERT bottle.

### Performance of the human mtDNA internal control

**Figure 3:**
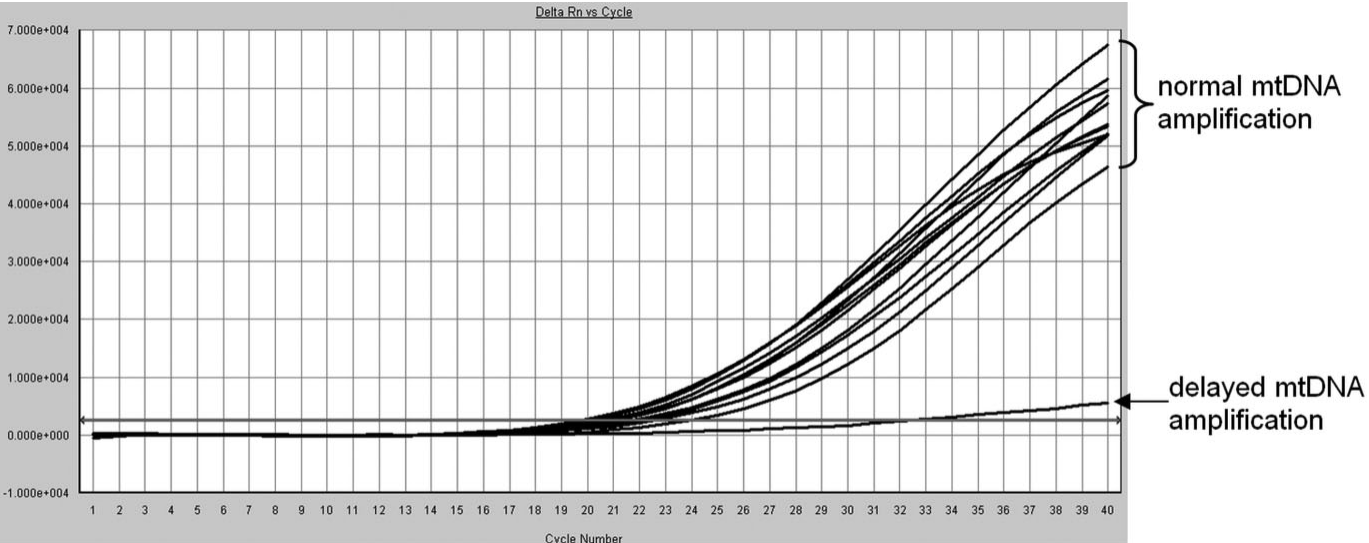
Example of delayed mtDNA amplification, in this case caused by competition from a very high *L. monocytogenes* 16S signal. The horizontal axis indicates cycle number and the vertical axis indicates fluorescence (Delta Rn).

To confirm efficient nucleic acid extraction, efficient amplification, and absence of PCR inhibitors, human mtDNA was coamplified in each reaction and served as an internal control. Ct values generated by the mtDNA PCR typically fell within the range 19 to 24 cycles. Delayed mtDNA amplification signals (>mean Ct + 3.5 SD) were observed in approximately 0.5% of the 2626 PLT concentrates tested. In every instance (other than delay caused by competition from very high 16S PCR signals; see Fig. 3) in which a delayed mtDNA amplification signal was observed, the PCR was repeated on the original nucleic acid extract and the mtDNA signal returned to within the expected Ct range.

## DISCUSSION

The behavior of the EMA-enhanced 16S rDNA real-time PCR assay in this present study was essentially the same as reported previously.^24^ However, the observation that PLT packs commonly contain trace amounts of bacterial DNA (Fig. 1B) necessitated increasing the assay threshold level to 25 genome copies per PCR procedure for the purposes of PLT screening. This low-level bacterial DNA, referred to here as “background noise,” is thought to be due to bacterial DNA fragments or nonviable organisms because it was present at the same level in both fresh and time-expired PLT concentrates. If viable organisms had been responsible, the “background noise” would have been expected to be higher in time-expired concentrates that had been stored at 22°C for 7 days. The presence of bacterial DNA fragments, detected by 16S rDNA PCR, in blood from healthy individuals has been noted previously.^26^,^27^

The results obtained from screening 2050 time-expired PLT packs by EMA-enhanced 16S rDNA real-time PCR were in agreement with those generated by BacT/ALERT in that no sample was confirmed positive in either assay. The observed low prevalence of bacterial contamination (<0.048%) in these PLT concentrates is consistent with recent reports from other countries^28^,^29^ and confirms the efficacy of improved donor skin disinfection and diversion techniques.^30^

Not surprisingly, the prevalence of confirmed bacterial contamination in the initial-reactive PLT concentrates (17 of 400 [4.2%] by BacT/ALERT) was significantly higher than in the time-expired PLTs. A wide range of incubation times (3.6-132 hr) was required before the BacT/ALERT automated culture system flagged these PLT concentrates as reactive. It is noteworthy that the three packs with the shortest incubation times (all ≤ 10 hr) were the three that were also detected by the 16S rDNA real-time PCR assay and are likely to have been the PLT packs with the highest bacterial concentrations.

The remaining 14 PLT concentrates, confirmed positive by BacT/ALERT but undetected by PCR required longer incubation times (associated with lower bacterial concentrations and/or more slowly replicating organisms) before being flagged as reactive. With the exception of *S. pneumoniae*, the organisms isolated from these PCR-negative packs are considered unlikely, especially at such low concentrations, to have resulted in significant harm to recipients.^31^–^33^ However, it is important to acknowledge 1) that the assumption of low bacterial concentration in such packs is based on inference rather than on direct measurement and 2) that our opinion concerning the improbability of harm to recipients in such cases is less certain for *S. salivarius* and *S. oralis* than it is for *P. acnes* and *S. saccharolyticus*, which have been shown not to proliferate in PLT packs.^31^–^33^ Reassuringly, for coagulase-negative *Staphylococcus*, Koopman and colleagues^32^ have recently reported that none of 33 recipients transfused with contaminated PLT packs experienced adverse reactions. Due to the long incubation periods (9 of 14 required > 54 hr incubation) and the policy of release on a “negative-to-date” basis, contaminated PLT packs such as these may be transfused before being flagged as reactive by BacT/ALERT, although this did not occur in this study.

*S. pneumoniae* is potentially highly pathogenic and it is therefore important to understand why it was not detected by 16S PCR in this case. There was no evidence of primer or probe sequence mismatch because the subcultured isolate was readily detected by the PCR assay in subsequent experiments. In our previous studies^24^ we demonstrated that the lower limit of detection by 16S PCR for *S. pneumoniae* is approximately 10 CFUs/mL and that the corresponding incubation time for BacT/ALERT at this level is approximately 13 hours. It therefore seems probable that the bacterial concentration in the contaminated pack was just below the limit of detection by PCR because the time taken for BacT/ALERT to flag reactive was 14.2 hours in this instance.

It is clear from these findings and from the findings of earlier studies^24^,^34^ that the analytical sensitivity of 16S PCR is lower than that of the BacT/ALERT automated culture system. The sensitivity difference is assumed chiefly to be due to the 40- to 80-fold greater sample volume used by the BacT/ALERT system (8 mL per culture bottle vs. 0.2 mL per PCR procedure, thus 80-fold if both aerobic and anaerobic cultures are performed) and to the fact that organisms can replicate in culture bottles for several days until they become detectable. The inability to run the PCR assay with a low detection threshold because of the presence of bacterial DNA fragments in blood from healthy donors further increases the sensitivity difference between the two assays. However, the lower sensitivity of 16S PCR may not be a problem in practice because, being a rapid assay (∼4 hr), testing can be delayed so that bacterial growth on PLT storage would potentially negate the sensitivity disadvantage. Rapid 16S PCR assays such as the one described here may therefore be more suited to a strategy of late or prerelease testing as proposed by Dreier and colleagues.^13^ The feasibility of such a late testing strategy in which a rapid real-time PCR-based assay would be employed after an approximately 48-hour holding period to allow bacterial replication has recently been demonstrated.^9^ This strategy, in principle, would allow PLTs to be issued as “negative” instead of negative to date.^34^ Alternatively, a prerelease strategy could be employed in which PCR-based testing would be performed immediately before transfusion, but the current approximately 4-hour duration of the assay would have to be reduced considerably to make this a practicable option. Such strategies could help reduce unnecessary recalls because organisms that do not proliferate in stored PLTs (e.g., *P. acnes* and *S. saccharolyticus*) would not be detected.^31^,^33^

In conclusion, this study confirms that EMA treatment is an effective solution to the previously intractable problem of bacterial DNA contamination in PCR reagents and that an EMA-enhanced real-time 16S PCR assay is capable of screening PLT concentrates without generating false-positive results. The study also demonstrates clearly that the sensitivity of such PCR assays is insufficient for use in early testing strategies, that is, as an alternative to automated culture systems such as BacT/ALERT. Future studies are being planned to evaluate the efficacy of this EMA-enhanced real-time 16S PCR assay in late or prerelease testing strategies.

## Abbreviations

Ct: threshold cycle
EMA: ethidium monoazide
mtDNA: mitochondrial DNA
NHSBT: National Health Service Blood and Transplant
NTC(s): no-template control(s)
